# The Experience of the Nursing Licensure Examination Among Newly Graduated Nurses: A Qualitative Study

**DOI:** 10.3390/nursrep15100347

**Published:** 2025-09-24

**Authors:** Flavia Pantaleo, Chiara Mastroianni, Michela Piredda, Alessandro Stievano, Natascia Mazzitelli, Laura Iacorossi, Maria Grazia De Marinis, Anna Marchetti

**Affiliations:** 1 Department of Biomedicine and Prevention, University of Rome Tor Vergata, 00133 Roma, Italy; 2 Department of Life Science, Health, and Health Professions, Link Campus University, Casale di San Pio V ST, 44, 00165 Rome, Italy; c.mastroianni@unilink.it (C.M.); l.iacorossi@unilink.it (L.I.); 3 Research Unit Nursing Science, Department of Medicine and Surgery, University Campus Bio-Medico, Alvaro del Portillo ST, 21, 00128 Rome, Italy; m.demarinis@policlinicocampus.it (M.G.D.M.); a.marchetti@policlinicocampus.it (A.M.); 4 Department of Clinical and Experimental Medicine, University of Messina, 98122 Messina, Italy; alessandro.stievano@unime.it; 5 Centre of Excellence for Nursing Scholarship OPI of Rome, Degli Ammiragli ST, 67, 00136 Rome, Italy; natascia.mazzitelli@aslroma5.it

**Keywords:** bachelor’s degree, evaluation of nursing competences, newly graduated, nursing licensure, nursing experience

## Abstract

**Background**: The nursing licensure examination is the final assessment of the university curriculum, certifying that students have acquired the competencies necessary for practicing the profession. Understanding the meaning and usefulness attributed to this test can contribute to the international debate on its value and potential continuation. In Italy, most studies in this field involve Directors of Degree Courses, while research on the lived experience of newly graduated nurses in facing the examination is lacking. **Objective**: The objective of this study is to explore the lived experience of newly graduated nurses during the licensure examination. **Methods**: A qualitative phenomenological study was conducted. Video–audio-recorded interviews were conducted and analyzed using Giorgi’s descriptive method, inspired by Husserl’s philosophy. **Results**: Fifteen nurses participated. The thematic analysis of the interviews revealed four significant areas: preparation and support received, ambivalent experience of the exam, emotions experienced during the exam, and the symbolic value ascribed to the exam itself. Each thematic area was further articulated into subthemes, for a total of ten analytical subdimensions. The licensure examination holds multiple meanings for new graduates: an opportunity for verification, a rite of passage, but also an emotionally charged event. While it represents a fundamental moment for constructing a professional identity, it also requires critical reflection on assessment methods, the fairness of the system, and the true educational value of the examination. **Conclusions**: Understanding the elements that influence the examination experience can help educators improve student preparation and promote a smoother transition to the professional role.

## 1. Introduction

The nursing licensure examination represents a milestone in the educational and professional trajectory of nursing students. It is not only a legal prerequisite for registration and practicing the profession [1,2] but also a moment of strong symbolic significance. As the concluding phase of academic training, the exam functions as a rite of passage, marking the transition from student to autonomous healthcare professional. It plays a key role in identity construction, formally recognizing the competencies and responsibilities of the newly graduated nurse [3], thereby contributing to professional identity and a sense of legitimacy within the nursing role.

At the international level, the organization and implementation of licensure examinations show considerable heterogeneity. Regulatory frameworks vary significantly across countries, resulting in a fragmented landscape in terms of exam structure and the evaluation of nursing competencies [4,5,6,7]. While some systems have developed standardized, competency-based assessment models, others still face challenges due to ongoing reforms [2]. Recurring issues include the lack of shared standards, limited clarity in assessment criteria, variability in the roles of clinical tutors and exam boards, and high levels of psychological and emotional stress experienced by candidates [8,9,10]. These difficulties are often exacerbated by institutional and contextual factors, such as the availability of human and material resources, the organization of nursing programs, and the degree of integration between academic and clinical learning environments [11].

The literature has addressed these challenges by promoting competency-based assessment approaches. Several studies have proposed frameworks aimed at aligning licensure examinations with professional standards and the clinical reasoning skills essential for safe and effective practice [10,12,13]. Attention has also been paid to graduates’ perceptions of preparedness, confidence during the transition to clinical practice, and the emotional and logistical challenges encountered during the examination process [3,14,15,16,17,18]. Other contributions have highlighted the psychological and structural obstacles faced by candidates, such as performance anxiety, fear of failure, and the pressure associated with exam outcomes [19,20,21].

In Italy, the nursing licensure examination is regulated by Ministerial Decree 270/2004 [1], which defines its structure and objectives. The exam consists of two components: a practical test, aimed at verifying the professional competencies acquired during clinical training, and a thesis defense, which serves as a moment of synthesis and critical reflection on the student’s educational journey [1]. Beyond its legal value, the examination formally marks the transition from student to professional, conferring identity and responsibility on those entering the healthcare workforce. The importance of the licensure examination is evident when considering recent data. In 2024, approximately 14,500 students graduated in nursing from over 50 accredited training institutions across Italy [22], compared to approximately 21,250 places available in undergraduate nursing programs, with an application-to-seat ratio ranging between 1.0 and 1.4 [23]. The pass rate varies by university but is generally estimated between 65% and 85%, highlighting inconsistency among institutions in terms of educational standards and assessment criteria [9,10]. Despite the important regulatory developments introduced with the Bologna Process in 1999—which harmonized university education across the European Higher Education Area through the adoption of a three-cycle academic system [24]—the Italian scientific literature has primarily focused on the perspectives of Degree Program Directors and Coordinators. Their efforts were directed at defining a shared core curriculum and reducing disparities among institutions [9,25,26], underscoring the need to increase coherence in assessment processes and strengthen the fairness and reliability of the licensure examination on a national scale. Therefore, the Italian literature still lacks attention to students’ subjective experiences; the emotions felt, the meanings attributed to the exam, and the lived experiences of candidates are nearly absent in national publications [27,28]. This gap risks rendering invisible an essential component of the professionalization process: how future nurses perceive their entry into the world of clinical responsibility and patient care. Understanding the experiences of newly graduated nurses could provide valuable insights for rethinking the entire educational pathway, improving didactic strategies, such as tutoring, simulation, and stress management support, and promoting regulatory decisions that are more equitable and aligned with the real needs of future professionals [29,30].

The general aim of this study was to explore the experiences of newly graduated nurses regarding the licensure examination. Specifically, the objectives were to (1) explore the factors that, from the nurses’ perspective, contributed to making the licensure exam a positive or negative experience; (2) explore their views on the usefulness of the licensure examination; and (3) explore how they would have preferred the examination to be organized and experienced.

## 2. Materials and Methods

### 2.1. Study Design

A qualitative observational study was conducted, employing the descriptive phenomenological approach of Giorgi [31], based on Husserl’s philosophical framework [32,33]. This methodology allows the elicitation of experiences, lived realities, emotions, and judgments of study participants. It also enables the interviewer to enter the inner world of the interviewees as they narrate their experiences. It is particularly suited to capturing the richness and complexity of lived experience, offering new interpretative frameworks that may prove useful in both educational and regulatory domains. The data analysis involved five key phases: (1) data collection through interviews, (2) reading the data, (3) dividing the data according to attributed meaning, (4) elaborating the data in an orderly language, and (5) synthesizing and revealing the essential structure of the phenomenon, according to Giorgi’s method [31].

### 2.2. Sampling and Participants

In accordance with the methodological recommendations for qualitative phenomenological research, the sample size was defined according to the criterion of theoretical saturation, the point at which data collection no longer yielded new information and the responses became repetitive or redundant [34,35].

Purposive sampling was employed to ensure methodological rigor, transparency, and quality of the sampling process. Eligible participants were nurses who had obtained a nursing degree within the previous 3 years at one of the five university campuses included in this study, affiliated with the Order of Nursing Professions (OPI) of Rome; who had 0–3 years of professional experience; who were of Italian or foreign nationality, with knowledge of the Italian language; who were in service during participation in this study; and who had provided informed consent. The inclusion criterion for nurses with 0 to 3 years of professional experience is supported by the existing literature that identifies this time frame as a critical transition phase. During these early post-graduation years, nurses undergo significant identity consolidation, skill development, and retrospective reflection on formative experiences, making the licensure examination a still-relevant and impactful event [36,37].

The participants practiced nursing as freelancers or employees, both nationally and abroad. The exclusion criteria included the following: cognitive or linguistic impairments that could compromise the participant’s ability to engage meaningfully and consciously in the interview process.

All the participants were informed in advance about the study objectives, interview procedures, and ethical aspects. Participation was voluntary, and each interviewee signed an informed consent form in accordance with the ethical principles of research.

### 2.3. Data Collection

This study commenced in March 2024, after receiving Ethics Committee approval on 28 February 2024. Data collection through interviews was completed in May 2024, while data analysis, interpretation, and manuscript preparation continued until this study formally concluded in March 2025. Qualitative data were gathered through semi-structured interviews guided by a topic guide, including open-ended questions aimed at fostering free and in-depth narration of the participants’ experiences. The main questions included the following: “*Could you describe your personal experience regarding the licensure exam?*”, “*What were your perceptions and emotions during the preparation and completion of the exam?*”, “*Could you share aspects or circumstances that positively and/or negatively influenced your perception of the licensure exam?*”, “*How did you perceive the usefulness of the exam with respect to your future professional career?*”*, and, finally,* “*Would you like to add anything else?*”.

Interviews were conducted by one of the researchers (FP), a third-year PhD student in Nursing, Midwifery, and Public Health Sciences, with experience in interviewing nursing students gained through her role as an Academic Tutor in a Nursing Degree Program. The researcher had no prior personal or professional relationship with the participants, ensuring a neutral position in data collection.

The email addresses of potential participants were provided by the Academic Directors of the five University Centers involved. They were first contacted via email to assess their potential interest in participating in this study. Interested candidates were contacted by phone to schedule the interview’s date, time, and location. Before starting the interview, all the participants signed an informed consent form.

The interviews were conducted either face-to-face or remotely via Google Meet, according to the participants’ preferences, and were video–audio recorded. In both cases, the sessions were held individually in neutral settings—physical or virtual—depending on the depth and amount of information shared, allowing space for the spontaneous emergence of unplanned issues. For the online interviews, a private and distraction-free environment was arranged and agreed upon in advance with each participant to ensure confidentiality and optimize the interaction. The recent literature supports the methodological validity of video-based qualitative interviews, particularly in phenomenological designs [38].

The researcher confirmed the appointment logistics the day before. Prior to each interview, the primary researcher (FP), assisted by a second researcher (LI), who is an expert in qualitative methodology with a PhD, presented the study aims and main topics to be explored, encouraging the participants to share their experiences freely. Sampling was achieved through saturation, as is expected for qualitative studies [35]. The interviews were conducted until data saturation was reached—defined as the point when no new relevant information emerged and the content became repetitive [35]. Saturation was achieved starting at the twelfth interview. During each interview, the researcher periodically summarized the participant’s statements to ensure accurate and shared interpretation. A socio-demographic form was used to supplement the interview process. The form gathered information on each participant’s age, gender, university of origin, year of graduation, and current professional status, providing contextual background to support the interpretation of the qualitative data.

### 2.4. Data Analysis

To ensure participant anonymity and facilitate data analysis, the five University Centers included in this study were coded as the first five letters of the alphabet (A, B, C, D, and E), and each interview was assigned a progressive number from 1 to 15. Descriptive analysis of socio-demographic characteristics was conducted using frequencies and percentages for categorical variables and means with standard deviations for continuous variables. The full textual corpus underwent reflexive thematic analysis following Braun and Clarke’s approach [34,35], integrated with Giorgi’s descriptive phenomenological methodology [31]. Analysis was semantic, adhering closely to the participants’ explicit words and perspectives, and followed an inductive approach to allow meanings to emerge directly from the data.

Initially, FP conducted the interviews, while LI transcribed them verbatim, including annotations on pauses, nonverbal expressions, gestures, and emotions observed during interviews. Subsequently, FP listened to the audio and video recordings and read the transcripts thoroughly to gain a deep, immersive understanding of the material. The transcriptions followed a predefined coding key to ensure consistency and standardization. In the case of unintelligible audio segments, a third researcher (AM) reviewed the recordings. If ambiguity remained, those portions were excluded from analysis.

In the third phase, aimed at identifying units of meaning, the data were reread line by line to isolate and code textual segments consistent with the participants’ lived experiences regarding the licensure exam [31]. The fourth phase involved organizing and elaborating the data: the researchers FP, LI, and AM inductively developed initial codes, organizing material into thematic units using NVivo 14 software [39]. This allowed open, reflective, content-centered coding.

The breadth and depth of identified themes were discussed within the research team (FP, CM, MP, AS, NM, LI, AM, MGDM) to assess their relevance. Rich or complex themes were subdivided into subthemes to clarify their essence and structure. To further enhance methodological rigor, an external audit was conducted by two independent researchers (AS and MP) who were not involved in the data collection or initial coding. This process systematically verified all the codes and themes, ensuring the credibility and trustworthiness of the analysis. Discrepancies in coding or theme interpretation were resolved through structured discussions within the research team until consensus was reached. Moreover, the participants were occasionally re-engaged to clarify or elaborate on specific statements made during the interviews, allowing the researchers to confirm that the coding accurately reflected their intended meanings. This approach ensured that the participants’ perspectives were accurately represented in the final themes. Each theme was then defined and named based on its central meaning, with particular attention to the subjective experience of newly graduated nurses regarding the licensure exam.

In the fifth and final phase, regarding the synthesis and elaboration of the essential structure, analytic writing was used to build a coherent, empirically grounded narrative addressing the study aims. Direct participant quotes were included to support the validity and transparency of the results. The analysis was conducted by the primary interviewer (FP), in collaboration with two researchers (AM and CM), while three senior researchers with extensive qualitative research experience (AS, MP, MGDM) supervised all the phases. Discrepancies during analysis were discussed within the team until a unanimous consensus was reached [35].

### 2.5. Rigor

To ensure the methodological robustness, validity, and reliability of the findings, several strategies were employed in line with the criteria for qualitative research proposed by Lincoln and Guba [40] and reiterated by Polit et al. [33]. Specifically, data saturation was used to guarantee analysis completeness, i.e., the point at which no new relevant information emerged and the participant responses became redundant [35].

Credibility was further strengthened through data and researcher triangulation, reducing the risk of bias from a single interpretative perspective. We deliberately did not conduct member checking as, in descriptive phenomenology, this procedure may alter the authenticity of lived accounts by inducing retrospective reinterpretation. Additionally, to ensure transparency and traceability of the research process, an independent audit trail was conducted by two researchers (AS and MP) who were not involved in data collection or analysis. This audit systematically verified all stages of the process, from empirical production to result formulation, consolidating study reliability.

The detailed description of the research design, participant selection criteria, and data collection and analysis techniques enabled us to accurately assess the transferability of the findings, facilitating their application to similar and scientifically comparable contexts.

Finally, the study reporting was guided by the Consolidated Criteria for Reporting Qualitative Research (COREQ), enhancing the transparency and completeness of the methodological description (Appendix A) [41].

### 2.6. Ethical Considerations

This study was conducted in accordance with the ethical principles set forth by the Declaration of Helsinki [42] and in compliance with current Good Clinical Practice regulations. This research received approval from the Institutional Review Board of the Campus Bio-Medico University of Rome (Study NUR-LIC, protocol no. 31.24 CET2 UCBM, approved on 28 February 2024). All the participants received information about the study objectives, the voluntary nature of participation, and their right to interrupt the interview at any time or to decline answering any question(s) without any consequence. It was also clarified that no financial compensation would be provided for participation. All the participants signed an informed consent form prior to their interview, in accordance with ethical research standards.

Confidentiality of personal data was guaranteed. Sensitive information was pseudo-anonymized and removed from the transcripts and any other study documents. Video data were accessible only to the research team members, and all documentation was securely stored by the responsible researcher (FP) in protected environments inaccessible to third parties.

Qualitative analysis was performed using NVivo software, version 14 [39], installed on a password-protected personal computer to ensure data security and compliance with data protection regulations.

## 3. Results

The sample consisted of 15 newly graduated nurses, evenly distributed across five university centers (three participants per center). The socio-demographic characteristics and the descriptive statistics of the sample are summarized in Table 1 and Table 2. The interviews lasted an average of 15.27 min (SD ± 3.36; range: 9–20 min). All the participants responded voluntarily and openly.

**Figure 1:**
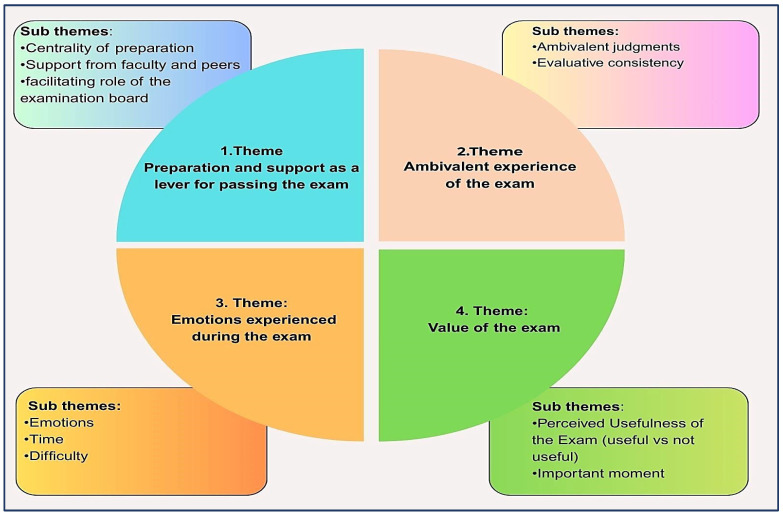
Themes and subthemes.

Four significant thematic areas were revealed that reflect the complexity, emotional ambivalence, and meanings attributed by the participants to the nursing licensure exam: preparation and support received, ambivalent experience of the exam, emotions experienced during the exam, and the symbolic value ascribed to the exam itself. Each theme area was further articulated into subthemes, for a total of ten analytical subthemes (Figure 1).

The presentation of the results is supported by selected verbatim excerpts, intended to convey the variety and depth of the experiences reported. The quotations are not merely illustrative but serve to highlight the tensions, convergences, and divergences within the narratives, thereby strengthening the interpretive framework. Each excerpt is accompanied by the alphanumerical code identifying each interview, composed of interview number (from 1 to 15), a letter representing the university center (from A to E), the participant’s gender (F/M), and the participant’s age at the time of the interview (e.g., 1A, F, 25). This system was adopted for descriptive and transparency purposes, without any direct influence on the analytic process.

### 3.1. Theme 1: Preparation and Support as a Lever for Passing the Exam

An important theme concerned the central role of preparation and the support received in shaping both the experience and outcome of the licensure exam. The participants acknowledged that facing the exam required a high level of commitment, and adequate preparation—both individual and guided—was perceived as a necessary condition to approach the test with confidence. This experiential theme is articulated through three subthemes: the centrality of preparation, support from faculty and peers, and the facilitating role of the examination board.

#### 3.1.1. Centrality of Preparation

All the participants acknowledged that the licensure exam required a high level of commitment and that thorough preparation was the only effective tool to tackle it. The exam was perceived as an opportunity to consolidate, systematize, and review all the knowledge and skills acquired during the three-year nursing program. Some of the participants emphasized that the exam encouraged them to engage in a comprehensive review, which they might not have undertaken otherwise. “*A negative aspect is the fact that you are forced to review all the topics covered over the three years, especially to memorize all the checklists completed from the first to the final year*” (3A, D, 24). “*With good preparation, you can definitely do it*” (5B, F, 32). “*The exam forces you to review all the activities, from the first to the last year of the degree course*” (11D, F, 24). “*It’s important; it may even prompt the student to do that complete review which, perhaps, wouldn’t have been done if it hadn’t been required for the state exam*” (15E, F, 24).

#### 3.1.2. Support from Faculty and Peers

Another facilitating element was the support received from both faculty members and fellow students. The participants provided contrasting evaluations of their ambivalent experiences during the licensure examination. Participants reported feeling accompanied and mentored throughout their academic journey, particularly during the preparation phase for the final exam. The support they received helped reduce anxiety and reinforced their sense of self-efficacy: “*If I hadn’t been so well supported, I don’t know if I would have graduated*” (12D, F, 22). “*The positive aspect is that the professors guided and mentored us the entire time; they gave us the opportunity to do a mock exam, and that gave us peace of mind, so we arrived at the exam well prepared*” (14E, F, 24).

Interaction with other students—both peers and those who had already graduated—was also perceived as an important source of reassurance. Peer dialogue allowed the participants to share uncertainties and expectations, helping them to approach the exam with greater calm: “*Let’s say mostly a lot of studying together with the other students and comparing thoughts about all the procedures that could possibly be on the exam*” (8C, F, 22). “*My colleagues who took it in previous years told me to stay calm… if you’ve studied so much for the internship exam, or if you’ve studied consistently throughout your degree, the licensure exam is a walk in the park*” (15E, F, 24).

#### 3.1.3. Facilitating Role of the Examination Board

The participants reported that the exam turned out to be less challenging than initially feared, thanks in part to the positive and reassuring attitude of the examination board. In many cases, the fact that board members were also course instructors contributed to creating a climate of trust and familiarity. The students felt listened to and put at ease, which helped reduce the emotional burden associated with being evaluated: “*The board listened with interest; that helped me feel calmer and more confident in explaining the various procedures I had to carry out. The board made us feel comfortable… I didn’t feel too much under pressure, even though there were many members lined up in front of me*” (8C, F, 22). “*Even though I didn’t give an outstanding performance, the board didn’t make things difficult for me. On the contrary, they helped and supported me. In the end, the exam was easier and quicker than I expected*” (11D, F, 24).

### 3.2. Theme 2: Ambivalent Experience of the Exam

The ambivalent nature of the experiences reported by participants during the licensure examination, generated contrasting evaluations. On the one hand, the interviewees acknowledged the coherence between the exam content and the three-year academic program; on the other, they pointed out a discrepancy in the emphasis placed on theoretical aspects over practical ones, which they considered essential to the nursing profession. Two subthemes articulated this experience: Ambivalent Judgments and Evaluative Consistency.

#### 3.2.1. Ambivalent Judgments

The participants described the structure of the exam, which was organized into two phases: a written component, consisting of the resolution of a simulated clinical case, and an oral component, which varied slightly depending on the training institution. The oral part included a discussion of nursing procedures randomly selected from a predefined list or their simulation on a mannequin: “*The exam is divided into two phases: a first phase, which involves explaining a pathology, and then identifying a nursing diagnosis and a collaborative problem*” (7C, M, 23). “*The practical part included all the checklists used in the laboratory exams throughout the three years, so a large number of practical procedures to be recalled in sequence, step by step*” (1A, F, 25). “*The oral part was held in the classroom with demonstrations on a mannequin*” (7C, M, 23). “*In my case, they also had me perform the mobilization of a patient with ALS, so it was a bit easier for me because I did the practical part on the mannequin while I was speaking*” (15E, F, 24).

“*The exam consists of a written test. This written test includes questions on all subjects, such as pharmacology and general and specialised nursing, scales and clinical cases, and … then those who pass it, those who get a good score on this test, can take the practical test … All of this increases anxiety … it would have been fair to have a final grade for both tests*” (3A, D, 24);

“*The clinical case seemed a bit banal to me. I don’t know if the test, the exam, is different for each university, but in my opinion, it wasn’t well designed to evaluate a nurse who has just started working or is about to start. It should be more complex and make you think, not remember.*” (2A, M, 22)

Although the participants recognized that the exam content was consistent with the material covered during their studies, several expressed concerns that the test placed greater emphasis on cognitive skills than on technical–practical competencies, which they considered essential for professional practice: “*In the end, it was a comprehensive review of everything we did over the three years*” (13E, F, 23). “*As for the practical part, let’s say there’s no other way to do it, but you can’t just memorize things and then not be asked to perform them in practice*” (2A, M, 22). “*The practical part, which for me was the most essential, seemed less important to them. I perceived this as a negative aspect*” (8C, F, 22). “*If the exam has to be done, more importance should be given to the practical part, to the simulation of activities, rather than making it mostly a verbal presentation of the content*” (12D, F, 22).

#### 3.2.2. Evaluative Consistency

Despite their criticisms, the participants acknowledged a certain degree of coherence between the exam content and what they had learned during their academic studies and clinical internship experiences. The questions and skills required during the exam were perceived as a logical and accurate extension of the training curriculum: “*The questions were clear and related to things we had already done during the internship; all the activities we prepared were the ones we had already studied during the three-year program, and the skills were those certified during the internship exams*” (10D, M, 30). “*Although there are many procedures to repeat and remember, they are still procedures that we performed correctly throughout the three years*” (15E, F, 24).

### 3.3. Theme 3: Emotions Experienced During the Exam

The participants reported experiencing intense emotions during the licensure exam. The emotions were complex and ambivalent, unfolding along a continuum between discomfort and satisfaction, tension and pride. The reported experiences highlight how the exam was not merely a technical and cognitive assessment but also a milestone laden with personal and professional significance. This theme is articulated into three subthemes: emotions, time, and difficulty.

#### 3.3.1. Emotions

The emotions most frequently described by the interviewees were anxiety, stress, agitation, and tension, experienced both during the preparation phase and throughout the exam. These feelings mainly stemmed from the fear of being insufficiently prepared or uncertainty regarding the exam format and the examination board’s expectations: “*The thought of failing one question and having to repeat the entire year is truly traumatic, so I felt a lot of anxiety*” (2A, M, 22). “*Personally, before the exam I felt anxious even though I had studied and was prepared, because you don’t know what kind of question you will get… so I was anxious, very tense…*” (10D, M, 30). “*I didn’t really enjoy that day… I remember the anxiety and stress more than the exam itself… in fact, I don’t even clearly recall the questions asked because I was so agitated and stressed*” (12D, F, 22).

In addition to anxiety, negative emotions, such as fatigue and disappointment, emerged. Fatigue was linked to the simultaneous management of the practical exam and thesis defense, often held on the same day:

“*It was difficult to manage the licensure exam and then, later, the thesis defense on the same day. We were exhausted… I was very tired psychologically*” (4B, F, 24). “*During preparation, I felt very tired … for me it was like a relief. I was eager for that day to come*” (6B, M, 28).

Disappointment was associated with the perception that the final evaluation did not reflect their effort:

“*In the end, they gave me a grade, but I didn’t like it. I wanted more … the board’s evaluation was not what I expected*” (11D, F, 24).

Alongside these negative experiences, positive emotions also emerged, such as joy, pride, and satisfaction for completing the educational path and obtaining professional licensure. Some of the participants highlighted a sense of self-efficacy and awareness of their own competencies: “*I was happy and proud because I had made it that far… the final emotion of passing the licensure exam and the graduation ceremony on the same day gave me so much joy*” (4B, F, 24). “*You feel confident, prepared… you know you are a competent person when you start working. That is the most important thing*” (3A, D, 24).

#### 3.3.2. Time

Time was perceived as a significant variable: seemingly endless during the preparation phase and extremely brief during the exam. This perceptual duality influenced how the experience was lived, intensifying the anticipatory effort and the swift nature of the evaluation moment:

“*At the beginning of preparation, time feels long, infinite… but during the written exam, time really flew by … the same goes for the checklists … in the end, it went by very quickly … a fleeting moment*” (3A, D, 24).

#### 3.3.3. Difficulty

The main difficulty highlighted by the participants was managing a large volume of theoretical and practical content alongside a thesis defense, often scheduled on the same day. Added to this was the discomfort of delivering an oral presentation in front of a large examination board, which heightened the sense of vulnerability: “*Studying all those 115 procedures and presenting the thesis made it harder for me to face the exam. Personally, I did not experience it very well*” (9C, M, 29). “*Explaining the diagnosis and intervention parts verbally in front of 10–12 people creates greater difficulty for us*” (7C, M, 23). “*Having to study and write the thesis simultaneously every day caused difficulties… then, entering the workforce immediately, I realized I could have handled it much more calmly*” (2A, M, 22).

Despite this, many participants emphasized that the exam was, in practice, less challenging than initially feared:

“*It was much easier than I thought. I expected it to be more complex*” (15E, F, 24). “*I expected a more difficult exam… but the level of difficulty was appropriate*” (12D, F, 22). “*The exam was easier than I expected*” (11D, F, 24).

### 3.4. Theme 4: Value of the Exam

The participants described the value attributed to the nursing licensure exam in ambivalent terms. On one hand, the exam was recognized as useful for reinforcing preparation, facilitating entry into the workforce, and promoting professional awareness; on the other hand, it was perceived as a formality with limited effectiveness in genuinely assessing competencies. This theme is divided into two subthemes: Perceived Usefulness of the Exam and Important Moment.

#### 3.4.1. Perceived Usefulness of the Exam (Useful vs. Not Useful)

Many of the participants considered the licensure exam useful for promoting a systematic review of all that was learned during the three-year training program. In particular, they emphasized the exam’s role in consolidating knowledge and skills, as well as encouraging a process of self-assessment regarding their level of preparedness:

“*For me, it is useful because you are forced to revisit all the topics covered over the three years, especially all the checklists you completed from the first to the last year*” (3A, D, 24). “*I think it is useful to understand how far you have progressed in your preparation… whether you have actually acquired the competencies you were supposed to*” (7C, M, 23).

Additionally, the exam was perceived as instrumental for entering the workforce, both as preparation for public sector job competitions and as an opportunity to facilitate integration within a professional team:

“*It was also very useful for work purposes and preparing to pass a public competition*” (1A, F, 25). “*It made me study again and helped me a lot in integrating into the work team*” (2A, M, 22).

However, other participants questioned its usefulness, considering it ineffective as a selective tool and poorly representative of actual professional competencies. For some, the exam appeared to be a bureaucratic formality, since competency evaluation already takes place during internships and assessments throughout the degree course: “*It’s useless as a filter between people ready to practice and those who are not*” (2A, M, 22). “*The licensure exam is useless from a training perspective because competencies are certified during the internship exams throughout the three years … it’s just repetition, a formality*” (9C, M, 29).

Building on this critique, several of the participants suggested structural changes to the exam, hoping for a streamlining of content, a greater uniformity in evaluation criteria across universities, and a better balance between theoretical and practical assessments: “*Maybe I would change the exam … there are 90 questions, many repetitive … I would combine some questions and streamline it*” (13E, F, 23). “*The exam is different for each university. In my opinion, it’s not well designed to assess a nurse starting work… the practical part is necessary, the one that forces you to reason*” (2A, M, 22).

#### 3.4.2. Important Moment

Regardless of their judgment of the exam’s usefulness, the participants unanimously recognized the symbolic and professional significance of the licensure test. For many, the exam represents a critical rite of passage that marks the transition from student status to entry into adult and professional life: “*The exam is important because it made me feel like a professional*” (6B, M, 28). “*At the end of the licensure exam, you go from being a student to an adult, you enter the adult world*” (9C, M, 29).

Moreover, the participants emphasized that the exam constitutes the sole formal and legal requirement for authorization to practice as a nurse, thereby conferring a social and professional legitimization function on the test: “*If you don’t pass the licensure exam, you’re not considered a nurse; it’s important so others can see you as a nurse*” (10D, M, 30). “*Not passing that exam means you are not authorized to practice the profession. This exam makes us nurses fortunate because, compared to other professionals, licensure for them requires further study after graduation*” (1A, F, 25).

## 4. Discussion

This study aims to explore the meaning that newly graduated nurses attribute to the experience of the licensure exam, with particular attention to the emotional, educational, and symbolic dimensions related to the transition from student to professional. Qualitative analysis identified four main themes and ten subthemes, reflecting the complexity of the exam experience in cognitive, emotional, organizational, and symbolic terms. This study offers a critical and in-depth understanding of the nursing licensure exam experience for newly graduated nurses, moving beyond a simple description to analyze the meaning attributed to this transitional phase. Although our findings confirm the importance of preparation and support—factors widely recognized in the literature as predictors of academic success [43,44,45]—they also provide a unique perspective on the Italian context. Unlike international systems, such as those in Anglo-Saxon countries, that use structured predictive exams to assess readiness [44,46], our participants described preparation as a self-directed, self-evaluation process, which raises questions about the effectiveness and equity of available preparatory methods.

The noted ambivalence toward the assessment reflects a broader debate on the need to balance the evaluation of theoretical knowledge with practical and relational competencies [47]. The participants’ frustration with the lack of realistic simulations and the marginalization of practical skills aligns with existing critiques that highlight how checklist-based evaluations may fail to capture clinical complexity [48,49]. Although the exam was perceived as consistent with the curriculum, this consistency does not necessarily translate into optimal preparation for professional practice. The integration of clinical judgment-based assessments [50] and the use of high-fidelity simulations could enhance the exam’s predictive validity, ensuring it not only tests memory but also anticipates real-world competence. Indeed, the emerging literature emphasizes the importance of assessment tools that reflect the decision-making process of clinical reasoning, rather than being limited to factual knowledge alone [51,52]. This approach represents a fundamental change in the very philosophy of assessment, aligning it with the complex demands of modern nursing practice.

This study also revealed that the perception of the exam as a “formality” or “redundancy” is a recurring theme among the participants, which contrasts with studies conducted in countries with standardized licensure systems. This divergence can be explained by several contextual reasons, including a lack of national standardization, which diminishes the exam’s symbolic value as an independent test of competence. Importantly, the theme of the patient relationship or clinical judgment in complex contexts emerged only marginally, unlike in other studies. The participants’ responses focused on managing performance anxiety and theoretical preparation, suggesting that the exam questions and related expectations may not have provided a sufficient opportunity to reflect on the nuances of patient care and the decision-making process. This interpretation is supported by recent research demonstrating that non-authentic assessment methods can limit students’ opportunities to showcase critical thinking and clinical judgment skills, influencing what students perceive as important for their learning and career [53,54].

Moreover, this study serves as empirical testimony to the Conservation of Resources Theory (COR) [55], demonstrating that support from faculty and peers not only mitigates stress but also acts as a buffer against the loss of emotional and psychological resources. The effectiveness of such systems can be further interpreted considering the role of professional resilience, defined as the ability to overcome challenges and grow professionally [56,57]. The availability of structured support, as found in our interviews, actively contributes to building this essential competence, preparing future nurses to navigate the complexities of their first year of practice. The recent literature indicates that resilience is not an innate trait but a skill that can be cultivated through training and support [58].

However, we recognize that implementing support systems, like tutoring, could pose an additional burden on students’ already limited time and energy. This concern, particularly relevant in the context of the Conservation of Resources Theory, suggests that poorly implemented support could inadvertently lead to further resource depletion rather than replenishment. To address this risk, academic institutions should not merely add optional tutoring programs but rather integrate resource-conserving strategies directly into the curriculum. This approach redefines support as an essential, not optional, component of the educational pathway, ensuring its benefits are equitably distributed among all students. This perspective is supported by recent research calling for the re-evaluation of how academic support is delivered to avoid compounding student workload and psychological stress [59].

The central role of time pressure emerged as a constant that, as suggested by Cognitive Load Theory [60,61], can compromise performance by reducing available mental resources. Finally, the ambivalent perceived value of the exam, which oscillates between being a “formal redundancy” and a “rite of passage,” along with the lack of national standardization, raises fundamental questions about its reliability and its role in ensuring equity in professional access [44,62]. This disparity not only compromises equity at the national level but also acts as an obstacle to the growing global movement for the mutual recognition of professional qualifications.

In summary, this study confirms the multidimensional complexity of the licensure exam, which carries multiple meanings for newly graduated nurses: assessment opportunity, rite of passage, technical test, and emotionally charged event. While fundamental to professional identity construction, it requires critical reflection on assessment methods, equity, and actual formative value.

### 4.1. Implications for Practice

The results of this study offer useful insights for improving the design and implementation of the nursing licensure examination, with significant implications for nursing degree programs as well as academic and regulatory institutions. First, the evidence highlighting the central role of systematic preparation and tutorial support suggests the need to strengthen structured student support pathways through educational activities that integrate academic tutoring, practical simulations, and progressive self-assessment tools. The introduction of standardized and progressive mock exams or predictive tests in the preparation phase for the final exam, as implemented in other international contexts [43,45,46], could assist students in monitoring their competence level prior to the final exam. Furthermore, alignment between the educational curriculum and assessment methods emerges as a crucial element. The findings underscore the importance of ensuring a genuine alignment between the competencies developed during the three-year program and those required during the exam, both in cognitive and practical–relational domains. This calls for reflection on the types of tests used, aiming for a more balanced distribution among theoretical, practical, and communication components, and greater attention to the authenticity of assessment situations, in accordance with recommendations for evaluating nursing competencies [63,64,65,66,67].

This research highlights a critical need to rethink the organization of nursing licensure exams. Data from the interviews and collected evidence reveal significant variations between universities, a serious concern that risks creating disparities among future professionals. We must address two key priorities: (a) Clarifying assessment objectives: What core competencies should we evaluate in newly graduated nurses? How can we assess these competencies objectively? (b) Implementing smart standardization: Standardization should not mean rigid uniformity. Instead, we need a flexible system that maintains high, consistent evaluation standards, adapts to different university contexts, and respects each institution’s available resources. As supported by the literature [63], this “intelligent standardization” approach ensures fair assessment practices, equal opportunities for all students, and consistency across universities. This framework moves beyond one-size-fits-all solutions, prioritizing both educational quality and real-world implementation.

### 4.2. Strengths and Limitations

The findings offer actionable insights for revising final preparation and assessment pathways, contributing to reflection on the formative, selective, and professional value of the current licensure model. In particular, this study highlights critical issues related to the coherence between expected competencies and adopted evaluation methods, underscoring the pivotal role of tutoring and peer support in exam preparation and emotional management—often overlooked aspects with significant impact that deserve greater emphasis in educational curricula. However, this study has some limitations that should be considered when interpreting the results. Like any qualitative study, the findings are not generalizable, but transferable [33].

The experiences collected may reflect organizational, cultural, and logistical specificities inherent to the involved institutions. The aim of this study was to explore newly graduated nurses’ experiences, but for a broader understanding of the phenomenon, the perspectives of other stakeholders should also be investigated. The absence of member checking may be viewed as a limitation; however, this was a conscious methodological choice to preserve the immediacy of the participants’ narratives within a phenomenological approach. Considering these findings, future research could broaden the perspective by exploring the experiences of newly graduated nurses from diverse geographic, academic, and cultural contexts—including international settings—to investigate convergences and divergences in experiences related to the licensure exam. Moreover, including the viewpoints of other stakeholders involved in the evaluation process, such as faculty members, clinical tutors, and examination board members, could provide a more integrated and systemic understanding of the exam experience. Finally, further exploration of the role of academic and peer tutoring represents a promising direction for developing more effective and humanizing educational interventions, capable of supporting students not only in technical preparation but also in emotional management and the assumption of professional responsibility during the delicate transition to nursing practice.

## 5. Conclusions

This qualitative study comprehensively explored the experiences of newly graduated nurses during the nursing licensure examination, revealing complex, multifaceted, and often ambivalent lived experiences. The four identified themes—preparation and support, ambivalent exam experience, experienced emotions, and the value of the exam—reflect the multiple meanings that the participants attributed to this crucial moment of professional transition.

Despite the critical issues related to the excessive emphasis on cognitive skills, variability in assessment methods across educational institutions, and the high emotional burden associated with the exam, the participants acknowledged the great symbolic and transformative value of the licensure examination. For many, it represents the completion of their educational journey and the beginning of their professional identity, marking the transition from student to recognized, competent, and socially reliable professional.

Thus, the exam is experienced not only as an evaluative test but as a rite of passage that confers legitimacy and awareness of one’s role. From this perspective, the value of the exam extends far beyond the technical-assessment dimension, assuming formative, identity-building, and motivational functions.

## Figures and Tables

**Table 1:** Characteristics of the participants.

Participant	Sex	Age	Nationality	High School	University Campus	Graduation Year	Graduation Time	Place of Origin	Worked During Degree	Work Experience		Employment Type	Country of Practice	Work Setting
										0–1 Year	2–3 Years			
1	Woman	25	Italian	Lyceums	A	2023	On time (3 years)	Puglia	No	Yes	No	Employee	Italy	Hospital
2	Man	22	Italian	Vocational School	A	2024	Extended (>3 years)	Lazio	Yes	Yes	No	Employee	Italy	Hospital
3	Woman	24	Italian	Vocational School	A	2023	Extended (>3 years)	Puglia	No	Yes	No	Employee	Italy	Hospital
4	Woman	24	Italian	Lyceums	B	2022	On time (3 years)	Lazio	No	Yes	No	Employee	Italy	Hospital
5	Woman	32	Italian	Lyceums	B	2022	On time (3 years)	Lazio	No	Yes	No	Employee	Italy	Hospital
6	Man	28	Italian	Vocational School	B	2023	On time (3 years)	Sicily	No	Yes	No	Employee	Italy	Hospital
7	Man	23	Italian	Lyceums	C	2023	On time (3 years)	Sicily	No	Yes	No	Employee	Italy	Hospital
8	Woman	22	Italian	Lyceums	C	2023	On time (3 years)	Sicily	No	Yes	No	Employee	Italy	Hospital
9	Man	29	Italian	Lyceums	C	2023	Extended (>3 years)	Sicily	No	Yes	No	Employee	Italy	Hospital
10	Man	30	Foreign	Lyceums	D	2023	On time (3 year)	Nigeria	No	Yes	No	Employee	Italy	Hospital
11	Woman	24	Foreign	Lyceums	D	2023	Extended (>3 years)	Lazio	No	Yes	No	Freelance	Italy	Home Care
12	Woman	22	Foreign	Lyceums	D	2023	On time (3 years)	Lazio	No	Yes	No	Employee	Italy	Hospital
13	Woman	23	Italian	Lyceums	E	2023	On time (3 years)	Calabria	No	Yes	No	Employee	Italy	Hospital
14	Woman	24	Italian	Lyceums	E	2023	On time (3 years)	Sicily	No	Yes	No	Employee	Italy	Hospital
15	Woman	24	Italian	Vocational School	E	2024	On time (3 years)	Puglia	No	Yes	No	Employee	Italy	Hospital

Legend: A = first university campus; B = second university campus; C = third university campus; D = fourth university campus; E = fifth university campus.

**Table 2:** Descriptive statistics of the sample (N = 15).

Variable	Category	Frequency (n)	Percentage (%)	Min	Max	Mean	Std Dev
**Gender**	Woman	10	66.7				
	Man	5	33.3				
**Age**				22	32	25.07	3.15
**Nationality**	Italian	12	80.0				
	Foreign	3	20.0				
**High School**	Lyceums Vocational School	11 4	73.3 26.8				
**University** **Campus**	A	3	20.0				
	B	3	20.0				
	C	3	20.0				
	D	3	20.0				
	E	3	20.0				
**Graduation Year**	2022	2	13.3				
	2023	11	73.3				
	2024	2	13.3				
**Graduation Time**	On time (3 years)	11	73.3				
	Extended (>3 years)	4	26.7				
**Place of Origin**	Puglia	3	20.0				
	Sicily	5	33.3				
	Nigeria	1	6.7				
	Lazio	5	33.3				
	Calabria	1	6.7				
**Worked During Degree**	Yes	1	6.7				
	No	14	93.3				
**Work** **Experience**	0–1 year	15	100				
	2–3 years	0	0				
**Employment Type**	Freelance	1	6.7				
	Employee	14	93.3				
**Country of Practice**	Italy	15	100.0				
	Abroad	0	0				
**Work Setting**	Hospital	14	93.3				
	Home Care	1	6.7				

Legend: N = sample; n = number of participants; % = percentage of participants; Std Dev = standard deviation.

## Data Availability

The data are contained within this article.

## Public Involvement Statement

There was no public involvement in any aspect of this research.

## Guidelines and Standards Statement

This manuscript was drafted against the COREQ (COnsolidated criteria for REporting Qualitative research) checklist for qualitative research [41].

## Abbreviations

The following abbreviations are used in this manuscript:

COR	Conservation of Resources Theory
COREQ	COnsolidated criteria for REporting Qualitative research
MIUR	Ministry of Education, University and Research
N	number of samples
n	number of participants
Std Dev	standard deviation

## Appendix A

**Table A1:** COREQ (COnsolidated Criteria for REporting Qualitative Research) Checklist [41].

Topic	Item No.	Guide Questions/Description	Reported on Page No.
**Domain 1: Research team and reflexivity**			
*Personal characteristics*			
Interviewer/facilitator	1	Which author/s conducted the interview or focus group?	Page 5
Credentials	2	What were the researcher’s credentials? E.g. PhD, MD	Page 5–6
Occupation	3	What was their occupation at the time of the study?	Page 6
Gender	4	Was the researcher male or female?	Page 6
Experience and training	5	What experience or training did the researcher have?	Page 6
*Relationship with participants*			
Relationship established	6	Was a relationship established prior to study commencement?	Page 6
Participant knowledge of the interviewer	7	What did the participants know about the researcher? e.g., personal goals, reasons for doing the research	Page 6
Interviewer characteristics	8	What characteristics were reported about the inter viewer/facilitator? e.g., Bias, assumptions, reasons and interests in the research topic	Page 6
**Domain 2: Study design**			
*Theoretical framework*			
Methodological orientation and Theory	9	What methodological orientation was stated to underpin the study? e.g., grounded theory, discourse analysis, ethnography, phenomenology, content analysis	Page 6
*Participant selection*			
Sampling	10	How were participants selected? e.g., purposive, convenience, consecutive, snowball	Page 5–6
Method of approach	11	How were participants approached? e.g., face-to-face, telephone, mail, email	Page 6
Sample size	12	How many participants were in the study?	Page 5–6
Non-participation	13	How many people refused to participate or dropped out? Reasons?	N/A
*Setting*			
Setting of data collection	14	Where was the data collected? e.g., home, clinic, workplace	Page 5–6
Presence of non-participants	15	Was anyone else present besides the participants and researchers?	Page 5
Description of sample	16	What are the important characteristics of the sample? e.g., demographic data, date	Page 7–8 (Table 1 and Table 2)
*Data collection*			
Interview guide	17	Were questions, prompts, guides provided by the authors? Was it pilot tested?	Page 5–6
Repeat interviews	18	Were repeat inter views carried out? If yes, how many?	N/A
Audio/visual recording	19	Did the research use audio or visual recording to collect the data?	Page 6
Field notes	20	Were field notes made during and/or after the interview or focus group?	Page 7
Duration	21	What was the duration of the inter views or focus group?	Page 5–6
Data saturation	22	Was data saturation discussed?	Page 5–6
**Domain 3: analysis and findings**			
*Data analysis*			
Transcripts returned	23	Were transcripts returned to participants for comment and/or correction	N/A
Number of data coders	24	How many data coders coded the data?	Page 5
Description of the coding tree	25	Did authors provide a description of the coding tree?	Page 6
Derivation of themes	26	Were themes identified in advance or derived from the data?	Page 5
Software	27	What software, if applicable, was used to manage the data?	Page 6
Participant checking	28	Did participants provide feedback on the findings?	N/A
*Reporting*			
Quotations presented	29	Were participant quotations presented to illustrate the themes/findings? Was each quotation identified? e.g., participant number	Page 11–12–13–14–15–16 Page 5–6
Data and findings consistent	30	Was there consistency between the data presented and the findings?	Page 11–12–13–14–15–16
Clarity of major themes	31	Were major themes clearly presented in the findings?	Page 11–12–13–14–15–16
Clarity of minor themes	32	Is there a description of diverse cases or discussion of minor themes?	Page 8,9,10,11–12,13,14,15–16
